# Supplementary material for: Health-related stigma among Indigenous Peoples in Canada: A scoping review
Source: PLoS One. 2025 Apr 21;20(4):e0318618. doi: 10.1371/journal.pone.0318618 (PMC12011227; doi:10.1371/journal.pone.0318618)
Supplement: S2 Table — This is the full search strategy as executed in MEDLINE. (DOCX) [file pone.0318618.s002.docx]

**S2. Full search strategy conducted in MEDLINE**

Ovid MEDLINE Search:

1. exp Social Stigma/

2. stigma*.mp.

3. health.ti,kf.

4. 1 or 2 or 3

5. (Canad* or British Columbia or Colombie Britannique or Alberta* or Saskatchewan or Manitoba* or Ontario or Quebec or (New Brunswick not New Jersey) or Nouveau Brunswick or Nova Scotia or Nouvelle Ecosse or Prince Edward Island or Newfoundland or Labrador or Nunavut or NWT or Northwest Territories or Yukon or Nunavik or Inuvialuit).mp,jw,nw. or (Abbotsford or Airdrie or Ajax or Aurora or Barrie or Belleville or Blainville or Brampton or Brantford or Brossard or Burlington or Burnaby or Caledon or Calgary or Cape Breton or Chatham Kent or Chilliwack or Clarington or Coquitlam or Drummondville or Edmonton or Fredericton or Fort McMurray or Gatineau or Granby or Grande Prairie or Sudbury or Guelph or Halton Hills or Iqaluit or Inuvik or Kamloops or Kawartha Lakes or Kelowna or Kingston or Kitchener or Langley or Laval or Lethbridge or Levis or Longueuil or Maple Ridge or Markham or Medicine Hat or Milton or Mirabel or Mississauga or Moncton or Montreal or Nanaimo or New Westminster or Newmarket or Niagara Falls or Norfolk County or North Bay or North Vancouver or Oakville or Oshawa or Ottawa or Peterborough or Pickering or Port Coquitlam or Prince George or Quebec City or Red Deer or Regina or Repentigny or Richmond or Richmond Hill or Saanich or Saguenay or Saint John or Saint-Hyacinthe or Saint-Jean-sur-Richelieu or Saint-Jerome or Sarnia or Saskatoon or Sault Ste Marie or Sherbrooke or St Albert or St Catharines or St John's or Strathcona County or Surrey or Terrebonne or Thunder Bay or Toronto or Trois-Rivieres or Vancouver or Vaughan or ((Cambridge or (Halifax or Hamilton or London or Victoria or Waterloo or Welland or Whitby or Windsor)) not (UK or Britain or United Kingdom or England or Australia)) or Whitehorse or Winnipeg or Wood Buffalo or Yellowknife).ti,ab,kw.

6. ((exp Indians, North American/ and Canad*.mp.) or Indigenous Canadians/ or exp Inuits/ or exp Health Services, Indigenous/ or (Athapaskan or Saulteaux or Wakashan or Cree or Dene or Inuit or Inuk or Inuvialuit* or Haida or Ktunaxa or Tsimshian or Gitxsan or Gitksan or "Nisga'a" or Haisla or Heiltsuk or Oweenkeno or "Kwakwaka'wakw" or "Nuu chah nulth" or "Tsilhqot'in" or Dakelh or "Wet'suwet'en" or Sekani or Dunne-za or Dene or Tahltan or Kaska or Tagish or Tutchone or Nuxalk or Salish or St'at'imc or Stl'atl'imx or Stl'atl'imc or Nlaka'pamux or Okanagan or "Sec wepmc" or Secwepemc or Tlingit or Anishinaabe or Blackfoot or Nakoda or Tasttine or "Tsuu T'ina" or "Tsuut'ina" or "Gwich'in" or Han or Algonquin or Nipissing or Ojibwa or Potawatomi or Innu or Maliseet or "Mi'kmaq" or Micmac or Passamaquoddy or Haudenosaunee or Cayuga or Mohawk or Oneida or Onondaga or Seneca or Tuscarora or Wyandot or Aboriginal* or Indigenous* or Metis or red road or "on reserve" or off-reserve or First Nation or First Nations or Amerindian).mp. or (urban adj3 (Indian* or Native* or Aboriginal*)).mp. or residential school*.mp. or autochtone*.mp. or (Native* adj1 (man or men or women or woman or boy* or girl* or adolescent* or youth or youths or person* or adult or people* or Indian* or Nation or tribe* or tribal or band or bands)).mp.) and (exp Canada/ or (Canad* or British Columbia or Colombie Britannique or Alberta or Saskatchewan or Manitoba or Ontario or Quebec or Nova Scotia or New Brunswick or Newfoundland or Labrador or Prince Edward Island or Yukon Territory or NWT or Northwest Territories or Nunavut or Nunavik or Nunatsiavut or NunatuKavut).mp.)

7. ((Aakom Kiyii or Alexis Cardinal River or Alexis Elk River or Alexis Nakota Sioux or Allison Bay or Assineau River or Atikameg or Athabasca Chipweyan or Beaver First Nation or Beaver Lake Cree or Bearspaw or Beaver Ranch or Bigstone Cree or Bistcho Lake or Blood Tribe or Cadotte Lake or Carajou Settlement or Chateh or Chiniki or Chipewyan Prairie or Cold Lake First Nations or Dene Tha or Desmarais Settlement or Dog Head or Driftpile or East Prairie or Elizabeth Settlement or Enilda or Enoch Cree or Duncans First Nation or Ermineskin or Fort Chipewyan or Frog Lake or Garden Creek or Goodfish Lake or Gregoire Lake or Grouard or Heart Lake or Hobbema or Hokedhe or Horse Lake* or Janvier Health or Jere Ghalil or Jean Dor or John Dor or Kainai or Kainaa or Kapaweno or Kee Tas Kee Now or Kehewin or Ki Tue or Kapaweno or Kikino or Kinuso or Jackfish Point or Little Buffalo or Little Red River Cree or Loon Prairie or Louis Bull or Lubicon Lake Indian Nation or Maggie Willier or Makaoo or Maskwacis or Meander Health or Mikisew or Nakota or Nakoda or Namur Lake or OChiese or Old Fort or Paddle Prairie or Paul Band or Paul First Nation or Peace Point or Peavine Settlement or Peigan or Peerless Lake or Peerless Trout or Piikani or Pikuni or Pikani or Puskiakiwenin or Red Earth Creek or Saddle Lake or Samson Cree or Sarcee or Sawridge Band or Siksika or Siksikawa or Sao-kitapiiksi or Stoney First Nation or Stoney Tribe or St Isadore or Sturgeon Lake Cree or Tasttine or Tallcree or Tall Cree or Thabacha or Thebathi or Tsuu Tina or Tsu Tue or Tsu Kadhe or Unipouheos or Utikoomak or Wabasca or Wesley Band or ((Alexander or Athabasca or Beaver Lake or Blue Quill* or Cold Lake or Duncans or Enoch or Fort McKay or Fort McMurray or Loon River or Paul or Smiths Landing or Sucker Creek or Sunchild or Swan River or Whitefish Lake) adj2 (First Nation* or tribe or Indian*))).mp. or (exp Indians, North American/ or exp Health Services, Indigenous/ or exp Medicine, Traditional/ or exp Shamanism/ or exp Ethnopharmacology/ or Indigenous*.mp. or Aboriginal*.mp. or Amerindian*.mp. or Autochtone*.mp. or First Nation.mp. or First Nations.mp. or Dene.mp. or Blackfoot.mp. or Anishinaabe.mp. or Assiniboine.mp. or Metis.mp. or Mischif.mp. or Mitchif.mp. or Metif.mp. or Metchif.mp. or Bois-brule*.mp. or Mixed-blood*.mp. or Half Breed*.mp. or halfbreed*.mp. or (traditional adj1 (medicine* or heal* or food* or health*)).mp. or Urban Indian*.mp. or "on reserve".mp. or "off reserve".mp. or country food*.mp. or shaman*.mp. or medicine m?n.mp. or medicine wom?n.mp. or ((native* or Indian or Indians) adj2 (person or persons or man or woman or men or women or child* or youth or youths or population* or people* or band or bands)).mp.)) and (Beaver Lake or Brownvale or Fort McMurray or Edmonton or Calgary or Hythe or Slave Lake or Valleyview or Fort Vermilion or Morinville or Glenevis or Lac La Biche or Cold Lake or Rocky Mountain House or Duffield or Brocket or Morley or Whitecourt or Amber River or Big Horn or Buck Lake or Charles Lake or Collin Lake or Cornwall Lake or Cowper Lake or Devils Gate or Eden Valley or Fox Lake or Little Red River or Hay Lake or Lesser Slave Lake or Bonneyville or Loon Lake or Wetaskiwin or Pigeon Lake or Lake Athabasca or Fort McLeod or Barrhead or Stony Plain or Sturgeon Lake or High Prairie or Swampy Lake or Upper Hay River or Wabamun or Trout Lake or Whitefish Lake or Winefred Lake or Nordegg or Boyer River or Calling Lake or Berwyn or Fort Chipewyan or Black Diamond or Fishing Lake or Gift Lake or Kananaskis or Medicine Hat or Ponoka or Stand Off or StandOff or Alberta).mp.

8. (((exp Indians, North American/ or exp Inuits/ or exp Health Services, Indigenous/ or exp Ethnopharmacology/ or (exp Medicine, Traditional/ not Chinese.mp.) or exp Shamanism/ or (Athapaskan or Saulteaux or Wakashan or Cree or Aboriginal* or Indigenous* or Metis or "off-reserve" or "on reserve" or First Nation or First Nations or Amerindian or (urban adj3 (Indian* or Native* or Aboriginal*)) or ethnomedicine or country food* or residential school* or shaman* or traditional medicine* or traditional heal* or traditional food* or medicine man or medicine woman or autochtone* or (Native adj1 (man or men or women or woman or boy* or girl* or adolescent* or youth or youths or person* or adult or people* or Indian* or Nation or tribe* or tribal or band or bands))).mp.) and (exp British Columbia/ or (British Columbia or Colombie Britannique or Williams Lake or Vernon or White Rock or Salmon Arm or Quesnel or Powell River or Port Moody or Port Hardy or Port Coquitlam or Port Albernie or Pitt Meadows or Penticton or New Westminister or Nanaimo or Kelowna or Kamloops or Fort St John or Fernie or Enderby or Dawson Creek or Coquitlam or Chilliwack or Campbell River or Tumbler Ridge or Skidegate or Sandspit or Queen Charlotte or Port Clements or Kitimat or Hudsons Hope or Haida Gwaii or Fort St James or Fort Nelson or Dease Lake or Pouce Coupe or Chetwynd or Gransile or Atlin or Alexis Creek or Sooke or Cache Creek or Chehalis or Cheslatta or Kingcome Inlet or Kitwanga or Iskut or Kyuqot or Kanaka or Clo-oose or Nicomen or Liard River or Mount Currie or Keremos or Matsqui or Nanaimo or Tahsis or Gitwinkshilkw or Osoyoos or Popkum or Sechelt or Skookumchuk or (Siska not Karol) or Barriere or Spuzzum or Sumas or Saanichton or Tsawwassen or Ucluelet or Quadra or Nemaiah Valley or ((Windermeer or Victoria or Vancouver or Terrace or Soda Creek or Surrey or Prince George or Prince Rupert or Parksville or Nelson or Merritt or Langley or Langford or Kimberley or Greenwood or Grand Forks or Duncan or Cranbrook or Courtenay or Colwood or Burnaby or Bonaparte or Armstrong or Abbotsford or Castlegar or Vanderhoof or Valemount or Stewart or Smithers or McBride or Massett or MacKenzie or Houston or Hazelton or Burns Lake or Fraser Lake or Alkali Lake or Ashcroft or Boston Bar or Spences Bridge or Port Douglas or Chase or Litton or Mill Bay or Lomcolith or D'Arcy or Sidney or Agassiz or Harrison Mills or Invermere or Telegraph Creek or Hope or Boothroyd or Trail) and Canad*)).mp.)) or (Duneza or Dunne-za or Dakelh or Babine or Wet'suwet'en or Haida or Sto:lo or Staulo or Stahlo or "Fraser River Indians" or Coast Salish or Kaska or Ktunaxa or Kootenay or Kwakwaka'wakw or Gitxsan or Gwich'in or Gwitich'in or Gitksan or Gwitchin or Gwichin or Kutchin or Tsimshian or Musqueam or St'at'imc or In-SHUCK-ch or Lil'wat or Lillooet or Nisga'a or Nuu-chah-nulth or Nootka or Nuxalk or Sekani or Wuikinuxv or Secwepemc or Sinixt or Skwxwu7mesh or (Tagish not meteorite) or Tahitan or Tahltan or Haisla or ((Nicola or Kitimat or Beaver or Okanagan or Sechelth) adj3 (man or men or boy or boys or girl or girls or child* or persons or adult* or youth* or adolescent* or Nation or people* or Indians* or tribe* or tribal* or band* or bands or person or population* or patient or patients or parent or parents or grandparent* or grandfather* or grandmother* or mother or mothers or father or fathers)) or (Carrier adj3 (Nation or Nations or Indians* or tribe* or tribal*)) or (Tsilhqot'in or Cowichan or Chilcotin or Nlaka'pamux or Tlingit or Tsetsaut or Oweekeno or Kwakiutl or Heiltsuk or Bella Bella or Saulteaux or Bella Coola or Shuswap or quamish or Stl'atl'imx or Stl'atl'imc or Stlatliumh or Slatemuk or Dane-zaa or Tsattine or Tutchone or Tuchone or Akisq'nuk or Esdilagh or Acho Dene Koe or Dene-tha or Adams Lake or Ahousaht or Aitchelitz or Beecher Bay or Blueberry River or Tsleil-Waututh or Burrard or Cacli'p or Canim Lake or Canoe Creek or Dog Creek or Cheam or Chawathil or Aishihi or Chehalis or Chemainus or Cheslatta or Comox or Da'naxda'xw or Ditidaht or Doig River or Dzawada'enuxw or Ehattesaht or Esketemc or Esquimalt or Gitanmaax or Gitanyow or Gitsegukla or Gitwangak or Gitxaala or Glen Vowell or Gwa'sala or Gwawaenuk or Hagwilget or Halalt or Halq'emeylem or Hesquiaht or Homalco or Hupacasath or Hul'quimi'num or Ka:'yu:'k't'h or Che'k'tles7et'h or Katzie or Kispiox or Kitselas or Kitsumlamun or Klahoos or Kluskus or Lhoosk'uz or K'omoks or Kwadacha or Kwaw-kwaw-a-pilt or Kwiakah or Kwicksutaineuk or Kwikwentlem or Lakahahmen or Lax-kw'alaams or Leq'a:mel or Lheidli-T'enneh or Lhatko or Lyackson or Malahat or Mamalilikulla or Matsqui or Metalakatla or Moricetown or Mowachaht or Muchalaht or Musqueam or Nadleh or Nak'azdli or 'Namgis or Sununeymuxw or Nanoos or Nazko or Nee-Tahi-Buhn or Neskonlith or Nisga'a or Nisgaa or Nooaitch or N'quatqua or Nuxalk or Ostlq'emeylem or Pacheedath or Pauquachin or Penelakut or Qayqayt or Quatsino or Saik'uz or Samahquam or Scowlitz or Semiahmoo or Shackan or Shxwha:y or Shw'ow'hamel or Simpcw or (Siska not Karol) or Skatin or Skawahlook or Skin Tyee or Skowkale or Skuppah or Skwah or Sliammon or Soowahilie or Spallumcheen or Squiala or Stellat'en or Taku River or T'it'qet or Tla-o-qui-aht or Tlatlasikwala or Tl'azt'en or Tl'etinqox-t'in or Tlowitsis or Toosey or Toquaht or Tsartlip or Tsawataineuk or Tsawout or Tsay Keh Dene or Tseshaht or Tseycum or Tsi Del Del or Ts'kw'aylaxw or Tsleil-Waututh or T'souke or Tzeachten or Uchcklesaht or Ulkatcho or We Wai Kai or Cape Mudge or Wuikinuxv or Xaxli'p or Yaakweakwioose or Yekooche)).mp.) not (isotope* or radiocarbon* or geology* or stratigraph* or Wisconsin or Michigan or Beaver County or (Alaska not (Alaska and (Canada or British Columbia)))).mp. not (animals not (humans and animals)).sh. [mp=title, book title, abstract, original title, name of substance word, subject heading word, floating sub-heading word, keyword heading word, organism supplementary concept word, protocol supplementary concept word, rare disease supplementary concept word, unique identifier, synonyms, population supplementary concept word, anatomy supplementary concept word]

9. (Opaskawayak or Little Saskatchewan or Fisher River Cree or Peguis or Sagkeeng or Roseau River or Norway House or Sapotaweyak or Wuskwi Siphik or Skownan or Dauphin River or Pinaymootang or Kinonjeoshtegon or O-Chi-Chak-Ko-Sipi or Tootinaowaziibeeng or Lake Manitoba or Keeseekoowenin or Waywayseecappo or Birdtail Sioux or Canupawakpa or Dakota Tipi or Brokenhead Ojibway or Northlands Nursing Station or Lac Brochet or Sayisi Cree or Tadoule Lake or Brochet or O-Pipon-Na-Piwin or South Indian Lake or Mathias Colomb or Pukatawagan or Tataskweyak or York Landing or Nisichawayasihk or Nelson House or Shamattawa or Bunibonibee or Manto Sipi or God's River or God's Lake or Red Sucker Lake or St Theresa Point or Wasagamack or Pauingassi or "Berens River" or Bloodvein).mp. or ((Sandy Bay or Long Plan or Sioux Valley or Fox Lake or War Lake or Pine Creek or Lake St Martin or Hollow Water or Little Black River or Rolling River or Dakota Plains or Swan Lake or Oxford House or Cross Lake or Split Lake or Barren Lands or Garden Hill or Poplar River or Little Grand Rapids).mp. and (exp Canada/ or Canad*.mp.)) or ((exp Indians, North American/ or exp Health Services, Indigenous/ or exp Medicine, Traditional/ or exp Shamanism/ or exp Ethnopharmacology/ or Indigen*.mp. or Aboriginal*.mp. or Amerindian*.mp. or Autochtone*.mp. or First Nation.mp. or First Nations.mp. or Metis.mp. or (traditional adj1 (medicine* or heal* or food* or health*)).mp. or Urban Indian*.mp. or "on reserve".mp. or "off reserve*".mp. or country food*.mp. or shaman*.mp. or medicine m?n.mp. or medicine wom?n.mp. or ((native* or Indian or Indians) adj2 (person or persons or man or woman or men or women or child* or youth or youths or population* or people* or band or bands)).mp.) and (exp Manitoba/ or Manitoba*.mp. or Winnipeg.mp. or Brandon.mp. or St Boniface.mp. or Dauphin.mp. or Flin Flon.mp. or Morden.mp. or Portage la Prairie.mp. or Selkirk.mp. or Steinbach.mp. or Thompson.mp. or Winkler.mp.)) [mp=title, book title, abstract, original title, name of substance word, subject heading word, floating sub-heading word, keyword heading word, organism supplementary concept word, protocol supplementary concept word, rare disease supplementary concept word, unique identifier, synonyms, population supplementary concept word, anatomy supplementary concept word]

10. (exp Indians, North American/ or exp Health Services, Indigenous/ or Metis.mp. or exp Medicine, Traditional/ or exp Shamanism/ or exp Ethnopharmacology/ or Indigenous*.mp. or Aboriginal*.mp. or Amerindian*.mp. or Autochtone*.mp. or Metis.mp. or First Nations.mp. or First Nation*.mp. or (traditional adj1 (medicine* or heal* or food* or health*)).mp. or Urban Indian*.mp. or "on reserve".mp. or "off reserve*".mp. or country food*.mp. or residential school*.mp. or shaman*.mp. or medicine m?n.mp. or medicine wom?n.mp. or ((native* or Indian or Indians) adj2 (person or persons or man or woman or men or women or child* or youth or youths or population* or people* or band or bands)).mp. or Montagnais.mp. or Maliseet.mp. or Naskapi*.mp. or Mi'kmaq.mp. or Micmac.mp. or Mic mac.mp. or Migmaw.mp. or Mig maw.mp. or Beothuk*.mp.) and (((Fredrickton or Moncton or New Jersey).mp. and (exp Canada/ or Canad*.mp.)) or exp New Brunswick/ or (New Brunswick* not ("New Brunswick NJ" or New Jersey or ferment*)).mp. or Nouveau Brunswick.mp. or Big Hole Tract.mp. or Metepenagiag.mp. or Eel Ground First Nation.mp. or Buctouche.mp. or Esgenoopetitj.mp. or Burnt Church.mp. or Devon Reserve.mp. or St Mary's First Nation.mp. or Eel River Reserve.mp. or Eel River Bar.mp. or Fort Folly Indian Point Reserve.mp. or Indian Island First Nation.mp. or Indian Ranch Reserve.mp. or Kingsclear.mp. or St John River Valley Tribal Council.mp. or Oromocto.mp. or Pabineau.mp. or Pokemouche.mp. or Mawiw.mp. or (Red Bank adj2 Reserve).mp. or Richibucto.mp. or St Basile.mp. or Madawaska.mp. or Soegao.mp. or Tabusintac.mp. or Tobique.mp. or Wolastoqiyik.mp. or Woodstock First Nation.mp.) [mp=title, book title, abstract, original title, name of substance word, subject heading word, floating sub-heading word, keyword heading word, organism supplementary concept word, protocol supplementary concept word, rare disease supplementary concept word, unique identifier, synonyms, population supplementary concept word, anatomy supplementary concept word]

11. ((exp Inuits/ or exp Indians, North American/ or exp Health Services, Indigenous/ or Medicine, Traditional/ or Shamanism/ or Ethnopharmacology/ or (Inuit* or Eskimo* or Esquimau* or Innu or Inuk or Innus or Metis or Montagnais or Maliseet or Naskapi or Mikmaq or Micmac or Mic mac or Migmaw or Micmaw or Beothuk* or Indigenous* or Aboriginal* or Amerindian* or autochtone* or First Nation or First Nations or Urban Indian* or on reserve* or off reserve* or country food* or medicine man or medicine men or medicine wom$n or shaman* or ethnomedicine* or ethnopharmacology).mp. or (Native adj3 (american* or man or men or women or woman or boy* or girl* or adolescent* or youth or youths or person* or adult or people* or Indian* or Nation or Nations or tribe* or tribal or band or bands)).mp. or (traditional adj1 (medicine* or heal* or food*)).mp.) and (exp "Newfoundland and Labrador"/ or (Newfoundland or Labrador or NFLD or Goose Bay or Sheshiatshiu or Nain or Rigolet or Hopedale or Utshimasset or Davis Inlet or Conne River or Postville or Makkovik or Lake Melville or Nunatsiavut or Urahimassit).mp.)) not (horse or pony or ponies or dog or dogs or puppy or puppies or canine or retriever*).mp.

12. ((((exp Medicine, Traditional/ not Chinese.mp.) or exp Shamanism/ or exp Indians, North American/ or exp Inuits/ or exp Health Services, Indigenous/ or exp Ethnopharmacology/ or (Inuit* or Eskimo* or Esquimau* or Athapaskan or Gwich'in or Metis or Inuvialuktun or Cree or Aboriginal* or Indigenous* or off-reserve or on-reserve or First Nation or First Nations or Amerindian or (urban adj3 (Indian* or Native* or Aboriginal*)) or ethnomedicine or country food* or residential school* or shaman* or traditional medicine* or traditional heal* or traditional food* or medicine man or medicine woman or autochtone* or treaty or (Native adj1 (man or men or women or woman or boy* or girl* or adolescent* or youth or youths or person* or adult or people* or Indian* or Nation or tribe* or tribal or band or bands))).mp.) and (exp Northwest Territories/ or Northwest Territories.mp. or NWT.mp. or Yellowknife.mp. or Western Arctic.mp.)) or (Aklavik or Banks Island or Behchoko or Rae Edzo or Colville Lake or De Cho or Deline or Denendeh or Fort Good Hope or Fort Liard or Fort McPherson or Fort McPherson or Fort Providence or Fort Providence or Fort Simpson or Fort Smith or Gameti or Hay River or Inuvik or Jean Marie River or Lutselk'e or Norman Wells or Paulatuk or Sachs Harbour or Trout Lake or Tsiigehtchic or Tuktoyaktuk or Tulita or Tulit'a or Ulukhaktok or Victoria Island or Whati or Wha Ti or Wrigley or (Hare adj2 (man or men or boy or boys or girl or girls or adult* or youth* or adolescent* or Nation or people* or Indians* or tribe* or tribal*)) or Slavey or Chipewyan or Tlicho or Dogrib or Yellowknives or Dene or Sahtu or Inuvaluit* or Inuinnaqtun).mp.) not ((fort smith adj1 ar*).mp. or ((rabbit* or lepus or lemming* or fox or foxes or wolf or wolves or (wrigley adj1 (n or g or forcep*))).mp. or ve.fs.)) [mp=title, book title, abstract, original title, name of substance word, subject heading word, floating sub-heading word, keyword heading word, organism supplementary concept word, protocol supplementary concept word, rare disease supplementary concept word, unique identifier, synonyms, population supplementary concept word, anatomy supplementary concept word]

13. (((((Indigenous* or Aboriginal* or Amerindian* or Autochtone* or First Nation or First Nations or (traditional adj1 (medicine* or heal* or food* or health*)) or Urban Indian* or "on reserve" or "off reserve*" or country food* or shaman* or medicine m?n or medicine wom?n or (native* or Indian or Indians)) adj2 (person or persons or man or woman or men or women or child* or youth or youths or population* or people* or band or bands)) or Montagnais or Maliseet or Naskapi* or Mi'kmaq or Micmac or Mic mac or Migmaw or Mig maw or Beothuk*).mp. or exp Health Services, Indigenous/ or exp Indians, North American/ or Metis.mp. or exp Medicine, Traditional/ or exp Shamanism/ or exp Ethnopharmacology/) and (((Wolfville or Middleton or Kentville or Berwick or Inverness or New Waterford or Sackville or Springhill or Halifax or Dartmouth or Truro or New Glasgow or Sydney or Canso or Guysborough or Parrsboro or Pictou or Liverpool or Lunenburg or Amherst) and Canad*).mp. or exp Nova Scotia/ or Nova Scotia*.mp. or Nouvelle Ecosse.mp. or Pictou Landing.mp. or Bear River.mp. or Boat Harbour.mp. or Annapolis Royal.mp. or Antigonish.mp. or Baddeck.mp. or Cheticamp.mp. or Cape Breton.mp. or Neil's Harbour.mp. or Glace Bay.mp. or Tatamagouche.mp. or Sheet Harbour.mp. or Cambridge Reserve.mp. or Annapolis Valley First Nation.mp. or Chapel Island First Nation.mp. or Cole Harbour.mp. or Eskasoni.mp. or Fisher's Grant.mp. or Franklin Manor.mp. or Paq'tnkek.mp. or (Glooscap adj1 (First Nation or reserve)).mp. or Acadia First Nation.mp. or Gold River Reserve.mp. or Horton Reserve.mp. or Shubenacadie First Nation.mp. or Indian Brook Reserve.mp. or Wagmatcook.mp. or Waycobah.mp. or Millbrook First Nation.mp. or Malagawatch.mp. or Medway River.mp. or Membertou.mp. or Merigomish.mp. or Musquodoboit.mp. or New Ross Reserve.mp. or Pennal Reserve.mp. or Pomquet.mp. or Poonhook.mp. or Sheet Harbour.mp. or St Croix Reserve.mp. or Summerside Reserve.mp. or Sydney Reserve.mp. or Truro Reserve.mp. or We'koqma'q.mp. or Wycocomagh.mp. or Wildcat Reserve.mp. or Yarmouth Reserve.mp.)) not (geology or geologic or stratigraphy* or animal* or cat or cats or kitten or deer or bird* or dog or dogs or feline or canine or bovine or equine or porcine or pig or piglet or swine or rat or rats or horse or horses or mouse or mice).mp. [mp=title, book title, abstract, original title, name of substance word, subject heading word, floating sub-heading word, keyword heading word, organism supplementary concept word, protocol supplementary concept word, rare disease supplementary concept word, unique identifier, synonyms, population supplementary concept word, anatomy supplementary concept word]

14. (exp Nunavut/ or Nunavut.mp. or Eastern Arctic.mp. or Alert Bay.mp. or Alexandra Fiord.mp. or Amadjuak.mp. or Aquiatulavik Point.mp. or Arctic Bay.mp. or Arviat.mp. or Baffin Island.mp. or Baker Lake.mp. or Bathurst Inlet.mp. or Belcher Islands.mp. or Bylot Island.mp. or Cambridge Bay.mp. or Iqaluktuttiaq.mp. or Cape Dorset.mp. or Cape Dyer.mp. or Cape Smith.mp. or Charlton Depot.mp. or Chesterfield Inlet.mp. or Clyde River.mp. or Coral Harbour.mp. or Craig Harbour.mp. or Dundas Harbor.mp. or Ellesmere Island.mp. or Ennadai.mp. or Eskimo Point.mp. or Fort Conger.mp. or Fort Hope.mp. or Fort Ross.mp. or Gjoa Haven.mp. or Grise Fiord.mp. or Hall Beach.mp. or Hazen Camp.mp. or Igloolik.mp. or Ikaluit.mp. or Iqaluit.mp. or Isachsen.mp. or Kekerten.mp. or Kimmirut.mp. or King William Island.mp. or Kipisa.mp. or Kitikmeot o r Kivalliq.mp. or Kivitoo.mp. or Kugaaruk.mp. or Kugluktuk.mp. or Maguse River.mp. or Nanasivik.mp. or Nottingham Island.mp. or Nuwata.mp. or Padlei.mp. or Padloping Island.mp. or Pangnirtung.mp. or Perry Island.mp. or Pond Inlet.mp. or Port Burwell.mp. or Qoloqtaaluk.mp. or Qikiqtarjuaq.mp. or Rankin Inlet.mp. or Read Island.mp. or Repuilse Bay.mp. or Resolute Bay.mp. or Resolution Island.mp. or Sanikiluak.mp. or Taloyoak.mp. or Tanquary Camp.mp. or Tavani.mp. or Thom Bay.mp. or Umingmaktok.mp. or Victoria Island.mp. or Wager Bay.mp. or Whale Cove.mp. or Eastern Arctic.mp. or ((Lupin or Polaris or Eureka or Fullerton) and Canad*).mp.) not (exp behavior, animal/ or exp ecosystems/ or exp endangered species/ or (sediment* or mantle or basalt* or cretaceous* or fossil* or paleo* or geolog* or stratigraph* or glaci* or refugia* or moraine* or pliocene or gravity or methylmercury or hydrolog* or hydrogeol* or volcan* or mesospher* or inferomet* or habitat* or animal behavior* or endangered species).mp.)

15. ((Aamjiwnaang or Pikwakanagan or (Animbiigoo adj Zaagi?igan adj Anishinaabek*) or Wauzhushk Onigum or Naongashiing or Anishnabekwe or Anishnawbe* or "ARMSTRONG SETTLEMENT" or Aroland or ASSABASKA or "Atikameksheng Anishnawbek" or Attawapiskat or "Aundeck-Omni-Kaning" or Batchewana or "Bearfoot Onondaga" or "Biinjitiwabik Zaaging" or Bimose or "Bingwi Neyaashi" or Bkejwanong or "chapleau cree" or dokis or Eabametoong or "Fox Lake Cree" or (moravian adj2 thames) or Ginoogaming or "Kasabonika Lake" or Kashechewan or KABAPIKOTAWANGAG or Keewaytinook or Kee?Way?Win or "Kiashke Zaaging" or Kitchenuhmaykoosib or Konadaha Seneca or Koocheching or Magnetawan or Matachewan or Mattagami or "MacDowell Lake" or M?Chigeeng or Mishkeegogamang or Missanabie or Mitaanjigaming or Stanjikoming or Mocreebec or (Mohawks adj (Akwesasne or Gibson)) or "Moose Cree" or Naicatchewenin or Namaygoosisagagun or Naotkamegwanning or Neskantaga or Nibinamik or Nigigoonsiminikaaning or Nipissing or OBADJIWAN or Obashkaandagaang or "Washagamis Bay" or Ochiichagwe Bibigo?ining or Onigaming or Parmachene or Sabaskong or Sagamok or (Oneida Nation adj2 Thames) or Shawanaga or Sheguiandah or Sheshegwaning or Taykwa Tagamou or Temagami or Wabaseemoong or Wabauskang or "Wabigoon Lake" or Wahgoshig or Wahnapitae or "Wahta Mohawks" or Wapekeka or Wasauksing or Wauzhushk Onigum or Wawakepewin or Webequie or Weenusk or Wikwemikong or ((Ardoch or Algonquin or Beausoleil or "big grassy" or "Iskatewizaagegan 39 Independent" or beaverhouse or "brunswick house" or "buffalo point" or caldwell or "cat lake" or Couchiching or "Curve Lake" or "deer lake" or delaware or "duck lake" or "Eagle Lake" or Thames or "Fort William" or "Garden River" or "Grassy Narrows" or Hiawatha or Henvey Inlet or Hornepayne or "Gull Bay" or "King Fisher" or (Lac adj Mille adj Lacs) or (Lac adj Croix) or "Long Lake" or Magnetawan or "Marten Falls" or "Martin Falls" or Mississauga or "New Credit" or "Moose Deer Point" or Munsee?Delaware or "Muskrat Dam" or "North Caribou Lake" or "North Spirit Lake" or Northwest Angle or Sioux Narrows or Pays Plat or Pic Mobert or "Red Rock" or "Sachigo Lake" or "Sandy Lake" or "Savant Lake" or Saugeen or Seine River or Serpent River or "Shoal Lake" or Stony Point or Stoney Point or "Grand River Territory" or "Slate Falls" or Whitefish River or Whitesand or "Whitewater Lake" or "Wunnumin Lake") adj2 first nation*)).mp. or (exp Indians, North American/ or exp Inuits/ or exp Health Services, Indigenous/ or exp Shamanism/ or exp Medicine, Traditional/ or exp Ethnopharmacology/ or American Native Continental Ancestry Group/ or (Peuple adj (autochtones or indidgenes or premier or racing or natif*)).mp. or ("Premiere Nation" or "First Nation" or "First Nations" or Metis or chippewas or Cree or Algonquin* or Algonquian* or Anishinabe* or Anishnabeg or Anishinaabe* or autochthon* or Inuit* or Innu or Innus or Innue or Micmac or Mic?Mac or Mi?gmaq or Mi?kmaq or Mowhawk or Ojibw* or Cayuga).mp. or (urban adj3 (Indian* or Native* or Aboriginal*)).mp. or ((Native not (bacteri* or plant* or species or micro* or biot* or strain or strains or probiotic or zoo* or geno*)) adj3 (american or man or men or women or woman or boy* or girl* or children or people* or indian* or Nation or tribe* or tribal or band or bands or groups or communit* or population* or health)).mp. or ((indigenous* not (bacteri* or plant* or species or micro* or biot* or strain or strains or probiotic or zoo* or geno*)) or Aboriginal* or (autochon* not (bacteri* or plant* or species or micro* or biot* or strain or strains or probiotic or zoo* or geno*)) or treaty or on-reserve or off-reserve or country food* or Shaman* or (traditional adj (medicine or heal*)) or residential school*).mp.)) and (exp Ontario/ or (ontario or toronto or ottawa or sarnia or london or hamilton or windsor or roseneath or "golden lake" or beardmore or "christian island" or "Cedar Point" or morson or "bay of quinte" or "bear island" or "Bearskin Lake" or "whitefish lake" or naughton or "little current" or "Kirkland lake" or "big island" or macdiarmid or "thunder bay" or wallaceburg or chapleau or leamington or "georgina island" or "CAPE CROKER" or "FOX LAKE" or "CHIEF'S POINT" or "Constance Lake" or "Curve Lake" or "deer lake" or "duck lake" or "Eagle Lake" or "ENGLISH RIVER" or Rama or Erie St?Clair or "FACTORY ISLAND" or "Flying Post" or "FORT ALBANY" or "FORT HOPE" or "Fort Severn" or "Fort William" or Wiarton or Southampton or Saugeen or Muncy or Calstock or "Fort Frances" or "FRENCH RIVER" or Thamesville or Monteville or "Eabamet Lake" or Nipigon or "Georgian Bay" or "Long Lac" or "Long Lake" or Grassy Narrows or Keene or Pickerel or Hornepayne or Glebe Farm or "GOULAIS BAY" or Grey? Owen Sound or GROS CAP or "Shoal Lake" or Kashechewan or INDIAN RIVER or "King Fisher Lake" or "Big Trout Lake" or "Sandy Lake" or "Fort Frances" or "Lac Seul" or "LAKE HELEN" or (LAKE adj WOODS) or Hudson or Britt or Okoki Post or "MANITOU RAPIDS" or gogama or "McDowell Lake" or "Red Lake" or Wawa or "New Osnaburgh" or "Garden River" or "Blind River" or Hagersville or "Port Perry" or "Moose Factory" or Cornwall or Deseronto or Mactier or Muncey or Delvin or Pawitik or "Whitefish Bay" or "Landsdowne House" or "Summer Beaver" or "Weagamow Lake" or "North Spirit Lake" or "Rainy Lake" or "Rainy River" or "Sioux Narrows" or Keewating or Kenora or "Sault Ste Marie" or "Nestor Falls" or "Heron Bay" or Southwold or "Pays Plat" or Mobert or "Pic River" or "Moon River" or Massey or "Sachigo Lake" or "Savant Lake" or Cutler or Nobel or Sheshegwaning or Kejick or Ohsweken or "Slate Falls" or Cochrane or "Lake Tamagami" or Thessalon or Whitedog or "Ear Falls" or Dryden or Matheson or Bala or Capreol or "Angling Lake" or "Parry Sound" or "Sioux Lookout" or Webequie or Peawanuk or "Birch Island" or Armstrong or "Wunnumin Lake" or Silverwater or Walpole Island or WINISK).mp.)

16. (exp Indians, North American/ or exp Inuits/ or exp Health Services, Indigenous/ or exp Shamanism/ or exp Medicine, Traditional/ or exp Ethnopharmacology/ or American Native Continental Ancestry Group/ or (Abenaki or Abenakis or Abitibiwinni or Akwesasne* or Atikamek* or "Barriere Lake" or Betsiamite* or Cacouna or Chisasibi or Coucoucache or "Eagle Village" or Eastmain or Essipit* or Ekuanitshit* or Gespeg or Gesgapegiag* or Huron-Wendat or "Huronne Wendat" or Essipit or Inuit or Inuk or Kahnawake or Kahnawa?ke or Kanesatake or Kawawachikamach* or Kebaowek or Kipawa or Kitcisakik or Kitigan Zibi or Kuujjuaq or "Lac Romanie" or "Lac John" or "Lac Simon" or Listuguj* or "Long Point First" or Maliotenam or Maliseet or Malecite* or Mamit Innuat or Mamuitun or Manawan or Mashteuiatsh or Matimekush* or Matimekosh* or Mawiomi or Migmaw or Mig Maw or Mi?gmawei or Mingan or Mistissini* or Montagnais or Naskapi* or Natashquan* or Nemiscau or Nemaska* or Obedjiwan or Odanak or Opitciwa* or dopitciwa* or Ouje?Bougoumou or Pakuashipi* or Pessamit* or Pikogan or "Rapid Lake" or Salluit or Schefferville or Sept-Iles or Takuaikan or "Uashat Mak" or "Mani-Utenam" or Temiscaming or imiskaming or Ungava or Uashat or "Unamen Shipu" or Waban-Aki or Waskaganish or Waswanipi or Wemindji or Wemotaci or Wendat* or Wendake or Whapmagoostui or Wolinak or Kitcisakik or "Pakua Shipu" or "Pakua Shipi" or Winneway or (Peuple* adj (autochtone* or indigene* or premier* or racine* or natif*)) or "Premiere Nation" or "First Nation" or "First Nations" or Metis or Cree or Algonquin* or Algonquian* or Anishinabe* or Anishinaabeg* or autochton* or Inuit* or Innu or Innus or Innue or Micmac or Mic?Mac or Mi?gmaq or Mi?kmaq or Mowhawk or (urban adj3 (Indian* or Native* or Aboriginal*)) or (Native adj3 (american or man or men or women or woman or boy* or girl* or children or people* or indian* or Nation or tribe* or tribal or band or bands or groups or communit* or population* or health)) or indigenous* or Aboriginal* or autochtone* or treaty or on-reserve or "off reserve" or country food* or Shaman* or (traditional adj (medicine or heal*)) or residential school*).mp.) and (exp Quebec/ or (montreal or "trois rivieres" or quebec* or "james bay" or "baie james" or Laval or Gatineau or Longueuil or Sherbrooke or Saguenay or Doncaster or Levis or Terrebonne or Mascouche or l?Estrie* or Lanaudiere or l?Outaouais* or Capitale-Nationale or Chaudiere-Appalaches or Cote-Nord or Gaspe* or Mauricie or Monteregie or Laurentides or Bas-Saint-Laurent or Saint-Laurent or Nunavik).mp. or (QC or quebec).in.)

17. ((Ahtahkakoop or Asimakaniseekan or Amisk Lake First Nation* or Assiniboine First Nation* or Beardy or Big River First Nation* or Birch Portage or Bittern Lake or Budd's Point or Mamawetan or Carry the Kettle or Canoe Lake or Carrot River or Chicken First Nation* or Kelsey Trail Health or Sunrise Health or Prince Albert Parkland Health or Day Star or Dipper Rapids or Eagles Lake First Nation* or Elak Dase or English River First Nation* or Prairie North Health or Fishing Lake First Nation* or Flying Dust First Nation* or Fond du Lac or Four Portages or Fox Point First Nation* or Athabasca Health Authority or Gordon First Nation* or Grandmother's Bay or Hatchet Lake or Hay First Nation* or Ils a la Crosse or James Smith First Nation* or Joseph Bighead or Kawacatoose or Kahkewistahaw or Key First Nation* or Kinistin or Kinookimaw or Kitsakie or Knee Lake First Nation* or La Loche First Nation* or La Plonge or Lac La Hache or Lac La Ronge or Little Black Bear or Little Bone First Nation or Little Hills First Nation* or Little Pine First Nation* or Little Red River or Lucky Man or Makaoo or Makaw or Meadow Lake First Nation* or Ministikwan or Minoahchak or Mirond Lake or Mistawasis or Montreal Lake First Nation* or Moosomin First Nation* or Morin Lake or Mosquito-Grizzley Bear's or Muscowpetung or Muskeg Lake First Nation* or Muskeg River First Nation* or Muskoday or Muskowekwan or Nekaneet or New Thunderchild or Cypress Health or Ocean Man or Ochapowace or Okanese or Okemasis or Old Fort First Nation* or One Arrow or Onion Lake or Opawakoscikan or (Pasqua not wheat) or Peepeekisis or Pelican Lake First Nation* or Pelican Narrows First Nation* or Peter Ballantyne or Peter Pond First Nation* or Pheasant Rump or Piapot or Potato River or Poundmaker First Nation* or Primeau Lake First Nation* or Red Earth First Nation* or Red Pheasant or Sakimay or Seekaskootch or Shesheep or Standing Buffalo or Starblanket or Tumor Lake or Wahpeton or Wapachewanak or Wa-Pii or Moos-Toosis or Waterhen or Willow Bunch or Witchekan or Wood Mountain or Yellowquill or Yellow Quill or (northern Saskatchewan not (uranium or selenium))).mp. or ((exp Indians, North American/ or exp Health Services, Indigenous/ or exp Medicine, Traditional/ or exp Shamanism/ or exp Ethnopharmacology/ or (Indigenous* or Aboriginal* or Amerindian* or Autochtone* or First Nation or First Nations or Metis or Michif or Urban Indian* or "on reserve" or "off reserve*" or country food* or residential school* or shaman* or medicine m?n or medicine wom?n or Buffalo River or Island Lake or Lean Man or Pine Bluff or Salteaux or Saulteaux or Sandy Narrows or Shoal Lake or Southend or Stanley or Sturgeon or Sucker River or Sweetgrass or White Bear or White Cap or Woody Lake or Cree or Dene or Chipewyan or Dakota or Algonquian).mp. or (traditional adj1 (medicine* or heal* or food* or health*)).mp. or ((native* or Indian or Indians) adj2 (person or persons or man or woman or men or women or child* or youth or youths or population* or people* or band or bands or reserve or reserves or treaty)).mp.) and (exp Saskatchewan/ or (Saskatchewan* or Sask or Regina or Estevan or Moose Jaw or Saskatoon or Lloydminster or Swift Current).mp.))) not (exp Medicine, Chinese Traditional/ or populus.mp. or India.mp. or Wisconsin.mp. or exp Plant Extracts/ or poplar.mp. or rats.mp. or veterinary.mp. or ve.fs.)

18. ((Carcross or (Tagish not meteorite*) or Champagne First Nation or Aishihik or Ehdiitat or Nacho Nyak Dun or Gwichya or Little Salmon or Carmacks or Nihtat or Selkirk First Nation or Ta'an Kwach'an or Tetlitn or Tr'ondek Hwech'in or White River First Nation or Vuntut or Yellowknives or (Hare adj2 (man or men or woman or women or child* or youth* or adult* or people* or person or persons or tribe or tribal or band or bands)) or Tanana or Tutchone* or Denesuline or Tahltan or MacKenzie Valley or Old Crow or "Upper Liard" or "Eagle Plains" or "Keno City" or Carcross or Teslin or "Fort Selkirk" or Carmacks or Haines Junction or Dawson City).mp. or ((Canad*.mp. or exp Canada/) and (Beaver Creek or Pelly or Destruction Bay or Watson Lake).mp.) or ((exp Indians, North American/ or exp Health Services, Indigenous/ or exp Medicine, Traditional/ or exp Shamanism/ or exp Ethnopharmacology/ or Indigenous*.mp. or Aboriginal*.mp. or Amerindian*.mp. or Autochtone*.mp. or Metis.mp. or First Nation.mp. or First Nations.mp. or exp Inuit/ or Inuit*.mp. or Chipewyan.mp. or Kaska.mp. or Kaskas.mp. or Tlingit.mp. or Dene.mp. or Gwich'in.mp. or Gwichin.mp. or Gwitchin.mp. or Kutchin*.mp. or Sahtu.mp. or Tlicho.mp. or Tli Cho.mp. or (traditional adj1 (medicine* or heal* or food* or health*)).mp. or Urban Indian*.mp. or "on reserve".mp. or "off reserve*".mp. or country food*.mp. or shaman*.mp. or medicine m?n.mp. or medicine wom?n.mp. or treaty.mp. or treaties.mp. or ((native* or Indian or Indians) adj2 (person or persons or man or woman or men or women or child* or youth or youths or population* or people* or band or bands)).mp.) and (exp Yukon Territory/ or Yukon*.mp. or ((Beaufort Sea or Whitehorse) and Canad*).mp.))) not ((exp Alaska/ or Alaska*.mp.) not ((exp Alaska/ or Alaska*.mp.) and (exp Yukon/ or Yukon*.mp.))) not (Yukon-Kuskok* or lepus or geology* or stratigraphi* or subduction* or volcan* or Holocene or pleistocene).mp.

19. or/6-18

20. 4 and 5 and 19

Note: Search filters were used for the Indigenous searches in Lines 6-18. All search filters can be found on this website: <https://guides.library.ualberta.ca/health-sciences-search-filters/indigenous-peoples>
