# Supplementary material for: Health-related stigma among Indigenous Peoples in Canada: A scoping review
Source: PLoS One. 2025 Apr 21;20(4):e0318618. doi: 10.1371/journal.pone.0318618 (PMC12011227; doi:10.1371/journal.pone.0318618)
Supplement: Attachment — Submitted filename: Response to reviewers.docx [file pone.0318618.s005.docx]

Response to Reviewers

Dear Editor

We appreciate the thoughtful suggestions from you and your reviewers. They have improved our manuscript. Below, we detail the revisions.

1. *Please ensure that your manuscript meets PLOS ONE's style requirements.*
   a. Completed.
2. *Please ensure that your PRISMA flow diagram is included in your main manuscript file as Figure 1.*
   a. We have had considerable difficulty positioning the PRISMA flow diagram in the main manuscript, so we have added it as a separate word document and identified its position in the main manuscript in the hope that the skilled staff at PLOS ONE can assist with this task.
3. *Please also provide non-author contact information (phone/email/hyperlink) for data access.*
   a. We have now included all of our data as Supplemental Information. The only data we did not submit originally was a table presenting all of the fulltext articles that were assessed for inclusion, along with the decision to include or exclude, and the data that was extracted from each of the included articles. That table is now available as an excel file named *S1 Fulltext articles assessed for inclusion*, and listed in the Supplemental Information section of the main manuscript.
4. *Please remove any funding-related text from the manuscript*.
   a. We removed the text from the manuscript and identified our funders appropriately within the Editorial Manager submission forms.
5. *Please include captions for your Supporting Information files at the end of your manuscript.*
   a. We have presented our titles and captions for our three supporting information files (p. 30), titled them appropriately, added captions, and updated the corresponding in-text citations correctly (p. 6-7).
6. *Please ensure your manuscript or supplementary information includes a numbered table of all studies identified in the literature search, including those that were excluded from the analyses. List the reason(s) for exclusion.*
   a. We included a table of all 997 studies assessed for inclusion, the decision that was made for each, by whom, when, and the data that was extracted (from the articles that were included). The table is included as a Supplement. (S3 Table. Articles assessed for eligibility.)
7. If applicable for your analysis, a table showing the completed risk of bias and quality/certainty assessments.
   a. Assessments of this nature are not required or conventional in *scoping reviews.* It is one quality that distinguishes scoping reviews from systematic reviews.
8. *Please review your reference list to ensure that it is complete and correct*.
   a. We reviewed and updated our reference list so that all in-text citations are included in the references list, the in-text citations are numbered consecutively, and the appropriate square-bracket format is used.
9. *The manuscript would benefit from additional references that contextualize the socio-cultural dimensions of stigma and its profound impact on the health outcomes of [Indigenous Peoples living in Canada].*
   a. In the final section of the Introduction (last paragraph of page 5) we added a brief discussion of the systematic racism and institutional stigma experienced by First Nations, Inuit, and Métis Peoples, and index the Canada’s Truth and Reconciliation Commission and the Canadian Medical Association’s 2024 apology for harms to Indigenous Peoples . We note too that our review focuses on the labelling, stereotyping, and discrimination that stems specifically from one’s health condition. This is in contrast to other discourses whose concern is the stigma that arises from gender, sexuality, ethnicity, a combination of these (i.e., intersectional stigma) or other factors.
10. *The inclusion of a PRISMA flow diagram would enhance the clarity of the methodological approach*.
    a. We have included our PRISMA flow diagram.
11. *The article would be strengthened by the inclusion of a conclusion section, a synthesis of key findings, and recommendations for future research in this area.*
    a. Pages 17 through 20 conclude the report, and they include i) a synthesis of key findings vis a vis our four research objectives in the 6 paragraphs following the heading *Discussion*. Suggestions for future research (and for practice) are presented in paragraphs 1 and 2 on page 19.
12. *I would like a bit more rationale for why you chose to focus on Indigenous Peoples in Canada, and not other countries.*
    a. Our study emphasizes the cultural nature of health-related stigma, and the Indigenous Peoples in Canada, comprising over 600 distinct communities, encompassing many different languages and ways of life that respond to remarkably different physical environments, presents an excellent opportunity to explore cross-cultural constructions of illness. At the same time, our selection bounded the study in ways that made it feasible and conceptually coherent.
13. *For international readers, you may want to include in the introduction about the inter-generational trauma experienced by this group and how this could impact both health and stigma and health-seeking behaviours.*
    a. We have implemented this important revision and described it in our response to a similar request in number point 9 (above). The new information is added to the stipulation in our original submission that:

       *“ . . . we excluded articles in which the participants’ experiences of stigma stemmed primarily from qualities other than their health condition; most frequently, these included the participants’ occupation, sexual orientation, gender, and cultural or ethnic identity. Stigma that arises at the intersection of these factors is a pressing topic for research; however, it was not the focus of this review.*”
14. *For the inclusion criteria, you stated you included primary research articles, but you did not stipulate whether you have any restriction on methods of the primary research articles (i.e. only qualitative articles)? If you did, this should be stated in the inclusion criteria.*
    a. We did not have any restrictions on “qualitative” or “quantitative” studies, and we have added a short qualification in the pertinent text in the Methods section. With the revision, it now reads: “We included only primary research (both post-positivist and interpretivist), and excluded articles categorized as essays, commentaries, position statements, letters, or reviews.”
15. *I would like a table title and number for all the tables in the manuscript.*
    a. *Table 2. Characteristics of included studies* (p. 10) lists all of the studies included in the manuscript, along with the relevant data that was extracted from each. Supporting Information *S3 Table Articles assessed for eligibility* presents the complete list of 997 articles that underwent fulltext screening.
16. *If some articles had a definition of stigma, this should be included in the data extraction. If none of them had a definition of stigma, this should be stated.*
    a. Some articles presented a definition of *stigma* and this was included in the data extraction. *S3 Table*, which is included for readers as supporting information, includes all of the data that was abstracted from the studies used in the scoping review, including the definitions of *stigma*.
